# Asymmetrical Neuroimaging Insights in CSF‐Validated Early‐Onset Alzheimer's: Integrating PET, MRI, and Cognitive Assessment

**DOI:** 10.1002/alz.094028

**Published:** 2025-01-09

**Authors:** Sinem Özçelik, Pınar Yiğit, Tolga Can Bozdemir, Gamze Çapa Kaya, Zerrin Yildirim, Boon Lead Tee, Hakan Gurvit, Görsev Yener, Maria Luisa Gorno Tempini, Didem Öz

**Affiliations:** ^1^ Dokuz Eylul University Hospital Neurology Department, Izmir Turkey; ^2^ Dokuz Eylul University Graduate School of Health Sciences, Izmir Turkey; ^3^ Dokuz Eylul University Hospital Department of Nuclear Medicine, Izmir Turkey; ^4^ Department of Neuroscience, Aziz Sancar Institute of Experimental Medicine, Istanbul University, Istanbul Turkey; ^5^ Memory and Aging Center, UCSF Weill Institute for Neurosciences, University of California, San Francisco, San Francisco, CA USA; ^6^ Behavioural Neurology and Movement Disorders Unit, Department of Neurology, Istanbul Faculty of Medicine, Istanbul University, Istanbul, Turkey, Istanbul Turkey; ^7^ Izmir Biomedicine and Genome Center, Izmir Turkey; ^8^ GBHI ‐ UCSF, San Francisco, CA USA

## Abstract

**Background:**

Early‐onset Alzheimer's disease (EOAD), manifesting before age 65, demands nuanced diagnostic approaches. FDG18‐PET unveils metabolic insights, the MRI scale captures structural changes, and ACE‐R assesses cognitive impairment details. A holistic evaluation enhances diagnostic precision and enriches our understanding of cognitive decline in early‐onset presentations.

**Method:**

Early‐onset Alzheimer's disease (EOAD), manifesting before age 65, demands nuanced diagnostic approaches. FDG18‐PET unveils metabolic insights, the MRI scale captures structural changes, and ACE‐R assesses cognitive impairment details. A holistic evaluation enhances diagnostic precision and enriches our understanding of cognitive decline in early‐onset presentations.

**Result:**

Twenty‐two patients (59.1% women) with a mean age at diagnosis of 55.3 (±6.52 StD) years participated. The mean disease duration was 5.06 (±2.95 StD) years. All of the patients were right‐handed. The mean Aß42 and phos‐tau were 392.49 (±200.13 StD) and 130.93 (±142.56 StD) pg/ml, respectively. No significant difference was found between the right and left insular cortex in PET, and no statistically significant asymmetry was found between VAS scores in MRI (p > 0.05). There was no correlation between PET index with CSF Aß42 and and phos‐tau values.

**Conclusion:**

This study underscores notable asymmetries in PET atrophy scores between the corresponding right and left brain regions in Alzheimer's patients. Given that EOAD manifests with atypical clinical symptoms, and all participants in this study exhibited left‐hemispheric dominance, the observed metabolic asymmetry, in the absence of a corresponding structural asymmetry, holds clinical significance.

